# Cognitive and gait in Wilson’s disease: a cognitive and motor dual-task study

**DOI:** 10.3389/fneur.2023.1243122

**Published:** 2023-08-31

**Authors:** Gongqiang Wang, Ping Jin, Xinfeng Ma, Xia Hong, Long Zhang, Kang Lin, Xiao Wen, Xue Bai, Yongzhu Han

**Affiliations:** ^1^Institute of Neurology, Anhui University of Traditional Chinese Medicine, Hefei, China; ^2^Affiliated Hospital of Institute of Neurology, Anhui University of Traditional Chinese Medicine, Hefei, China; ^3^Graduate School of Anhui University of Traditional Chinese Medicine, Hefei, China

**Keywords:** Wilson’s disease, cognitive impairment, gait disturbance, dual task, cognitive and gait

## Abstract

**Background:**

Cognitive and motor dual-tasks play important roles in daily life. Dual-task interference impacting gait performance has been observed not only in healthy subjects but also in subjects with neurological disorders. Approximately 44–75% of Wilson’s disease (WD) patients have gait disturbance. According to our earlier research, 59.7% of WD patients have cognitive impairment. However, there are few studies on how cognition affects the gait in WD. Therefore, this study aims to explore the influence of cognitive impairment on gait and its neural mechanism in WD patients and to provide evidence for the clinical intervention of gait disturbance.

**Methods:**

We recruited 63 patients who were divided into two groups based on their scores on the Addenbrooke’s cognitive examination III (ACE-III) scale: a non-cognitive impairment group and a cognitive impairment group. In addition to performing the timed up and go (TUG) single task and the cognitive and motor dual-task digital calculation and animal naming tests, the Tinetti Balance and Gait Assessment (POMA), Berg Balance Scale (BBS), and brain MRI severity scale of WD (bMRIsc-WD) were evaluated. The dual-task cost (DTC) was also computed. Between the two groups, the results of the enhanced POMA, BBS, and bMRIsc-WD scales, as well as gait performance measures such as TUG step size, pace speed, pace frequency, and DTC value, were compared.

**Results:**

(1) Among the 63 patients with WD, 30 (47.6%) patients had gait disturbance, and the single task TUG time was more than 10 s. A total of 43 patients had cognitive impairment, the incidence rate is 44.4%. Furthermore, 28 (44.4%) patients had cognitive impairment, 39 (61.9%) patients had abnormal brain MRI. (2) The Tinetti gait balance scale and Berg balance scale scores of patients with cognitive impairment were lower than those of patients without cognitive impairment (*p* < 0.05), and the pace, step size, and pace frequency in the single task TUG were slower than those of patients without cognitive impairment (*p* < 0.05). There was no change in the pace frequency between the dual-task TUG and the non-cognitive impairment group, but the pace speed and step size in the dual-task TUG were smaller than non-cognitive impairment group (*p* < 0.05). There was no difference in DTC values between cognitive impairment group and non-cognitive impairment group when performing dt-TUG number calculation and animal naming respectively (*p* > 0.05). However, regardless of cognitive impairment or not, the DTC2 values of number calculation tasks is higher than DTC1 of animal naming tasks in dt-TUG (*p* < 0.05). (3) Pace speed and step size were related to the total cognitive score, memory, language fluency, language understanding, and visual space factor score of the ACE-III (*p* < 0.05), and step frequency was correlated with memory and language comprehension factors (*p* < 0.05). There was no correlation between the attention factor scores of the ACE-III and TUG gait parameters of different tasks (*p* > 0.05). Brain atrophy, the thalamus, caudate nucleus, and cerebellum were correlated with cognitive impairment (*p* < 0.05), the lenticular nucleus was related to the step size, brain atrophy was related to the pace speed, and the thalamus, caudate nucleus, and midbrain were involved in step frequency in WD patients (*p* < 0.05).

**Conclusion:**

WD patients had a high incidence of cognitive impairment and gait disorder, the pace speed and step size can reflect the cognitive impairment of WD patients, cognitive impairment affects the gait disorder of WD patients, and the different cognitive and motor dual-tasks were involved in affecting gait parameters. The joint participation of cognitive impairment and lesion brain area may be the principal neural mechanism of gait abnormality in WD patients.

## Introduction

Wilson’s disease (WD) is a rare complex disorder of copper metabolism caused by ATP7B gene mutation, which leads to the unregulated accumulation of copper in various tissues. It usually takes place in adolescence, and its clinical manifestations are progressive neuropsychiatric symptoms, liver cirrhosis, renal damage, and Kayser–Fleischer rings. WD belongs to just a few genetic disorders which can be satisfactorily managed if diagnosed early and correctly treated. However, if left untreated, WD is universally fatal (1). According to studies, 44% ~ 75% of WD patients have aberrant gaits (2, 3), which has a significant effect on the quality of life, prognosis, and safety, especially in neurologic patients. For a very long period, gait disturbance in WD patients were frequently attributed to the clumsy symptoms such as Parkinson’s-like, tremor, dystonia, and chorea-like caused by extrapyramidal or cerebellar pathological changes (4).

In recent years, studies have confirmed that cognitive function is closely related to gait, and one’s cognitive state can directly affect one’s quality of gait (5). Gait requires complex synchronization at every level of the nervous system, yet occurs with minimal conscious effort on behalf of the walker. Furthermore, a growing body of data has demonstrated that gait abnormalities predict the future development of dementia, and cognitive impairment increases the risk of falls. Cognitive and motor dual tasks play important roles in daily life, and dual-task interference impacting gait performance has been observed not only in healthy subjects but also in subjects with neurological disorders (6). Gait disturbances are frequent in WD, and reflect the involvement of many brain structures (7). Our previous study found that 59.7% of WD patients had cognitive impairment (8). A review showed that cognitive and motor dual-task exergames in healthy older adults were more effective in improving physical and especially cognitive functions than single task or control conditions (9). Does cognitive impairment affect the gait disturbance of WD patients? At present, there are few studies on the influence of cognitive dysfunction on the gait of WD patients. This study focuses on the manifestations of and correlations between cognitive and gait impairment in WD, and provides guidance for an early clinical approach to intervene in them.

## Materials and methods

### Participants

A total of 63 WD patients, hospitalized in the Department of Neurology, Affiliated Hospital of the Institute of Neurology, Anhui University of Traditional Chinese Medicine, were selected according to the following inclusion criteria: (a) patients undergoing initial treatment and meeting WD diagnostic criteria (10) and (b) an age ≥ 14 years old, junior high school education or above, the ability to cooperate, and written informed consent provided for study participation. The WD patients with elevated blood ammonia and other diseases or drugs that affecting cognitive function and gait were excluded. The characteristics of the enrolled participants were shown in 63 patients were enrolled, including 29 males and 34 females, the age ranged from 15 to 45 years. In terms of educational level, there were 34 junior high school students, 18 senior high school students and 11 college graduates.

### Collection of general information

The patients’ information was collected by an original summary table of general data, including patients’ names, ages, gender, years of education, course of disease, main clinical manifestations, and so on. Patients were divided by binary clinical phenotype classification into hepatic type and neurologic type. Hepatic type was defined as hepatic presentation at the time of diagnosis of exclusively hepatic symptoms and signs and the absence of neurologic symptoms and signs as confirmed by careful neurologic examination. Neurologic type was defined as neurologic presentation and the absence of hepatic symptoms at the time of diagnosis (2).

### Neurological function scale evaluation

First, cognitive function was assessed, and then, after a 15 min rest, other neurological function scales were evaluated in turn. The cognitive function evaluation was conducted in a quiet and well-lit neuropsychological evaluation room, and the test time was arranged to start at 4 pm after normal lunch, and the project test and result evaluations were conducted by the same professionally trained neuropsychological surveyor in strict accordance with the scale instructions.

The Chinese version of Addenbrooke’s Cognitive Examination III (ACE-III) scale was used (11, 12). The scale evaluates cognitive impairment, with a total score of 100, and includes five factors: attention, memory, language fluency, language, and visual space. The higher the score, the better the cognitive function. In this study, referring to the literature, the total score of less than 88 points is classified as cognitive impairment group, and the score of more than or equal to 88 points is classified as non-cognitive impairment group.

The Tinetti Balance and Gait Assessment (POMA) (13) scale consists of two parts: a gait assessment table and a balance assessment table, of which the balance test has nine items, with a full score of 16, and there are eight items in gait test, with a full score of 12. If the overall score is less than 24, it means that there is balance dysfunction; if it is less than 15, it means there is a danger of falling.

The Berg Balance Scale (BBS) (14) scale consists of 14 items such as standing, supporting, and transferring, and each item has a score of 0–4, with a maximum score of 56, and the score is directly proportional to balance ability.

### Cognitive and motor dual-task gait tests

The experimental method of the 3-meter Timed Up and Go (TUG) and cognitive and motor dual-task gait tests were adopted (15, 16). The same objective was tested with a single task (st-TUG) and two dual tasks (dt-TUG) successively. The st-TUG test completes the task of standing up and walking on a trail according to instruction, in which if the test time is more than or equal to 10 s, the gait is judged to be abnormal (17). The dt-TUG1 task is to complete the calculation task of continuously subtracting 7 from 100 while completing st-TUG task, while the dt-TUG2 task is to complete the naming task of naming as many animals as possible without interruption while completing st-TUG task. In the test process, the gait performance parameters such as the walking speed, step length, and step frequency of patients are analyzed and extracted by using the gait capture timing method of three-dimensional photographic video. Using the formula of dual-task cost (DTC) (18), the values of DTC1 and DTC2 for executing the two different cognitive dual tasks are calculated, respectively. The definitions of each gait parameter extracted in the TUG experiment are shown in Table 1.

**Table 1:** Definition method of each gait parameter extracted in TUG experiment.

Gait parameter	Definition
Walking time (s)	Time taken to complete the TUG trail and turn back and go straight for 6 meters (excluding turning around)
Step count (*n*)	Steps taken to complete the TUG trail and turn back and go straight for 6 meters (excluding turning)
Step size (m)	Divide the 6 meters length of the straight trail by the step count, which is the average step size.
Pace speed (m/s)	Divide the 6 meters length of the straight trail by the walking time, which is the average pace speed.
Pace frequency (*n*)	Turn back to TUG Trail and go straight for 6m, the step count per minute is the average step frequency when going straight.
Dual-task cost (*n*)	(single task pace-dual task pace) ÷ single task pace × 100%

### Brain MRI examination and semiquantitative severity assessment

All the enrolled subjects underwent Siemens magnetic resonance imaging (MRI) 1.5T multi-scanner brain examination, the scanning sequence included T1-weighted, T2-weighted, diffusion-weighted imaging (DWI), susceptibility weighted imaging (SWI), and fluid-attenuated inversion recovery (FLAIR), recorded the pathological brain regions corresponding to each enrolled subject in detail, and the lesions in different brain regions of the same WD patient were recorded as one case. The brain MRI severity scale of WD (bMRIsc-WD) was used to evaluate the radiological severity of abnormal brain MRI (19).

### Statistical analysis

The data were analyzed using the SPSS statistical software package, Version 23.0 (IBM corp.). Subject characteristics at baseline and all primary and secondary outcomes were summarized using descriptive statistics. Data were tested for normality and homogeneity of variance and are summarized as mean and standard deviation. Two independent sample *t*-tests were used to compare the sample data between the two groups, and paired *t*-tests were used to compare the sample data within the group. An ANOVA was used to determine whether or not there was a statistically significant difference between the means of three or more independent groups. Pearman’s correlation coefficients were used to measure the correlation between gait parameters and cognitive domain factors. The stepwise method was used to analyze the correlation between various dual-task gait performance parameters of the TUG and cognitive factors and pathological brain regions in different task backgrounds of the TUG. The level of statistical significance was set at *p* < 0.05.

### Ethics statement

All procedures performed in the studies involving humans were in accordance with the ethical standards of the institutional and national research committee and with the 1964 Declaration of Helsinki and its later amendments. Informed consent was obtained from the patients or guardians. The study was approved by the local university ethical review authority.

## Results

### Distribution of cognitive impairment and gait abnormality

Among the 63 WD patients, there were 28 (44.4%) patients with cognitive impairment, including 10 males and 18 females, depending on the results of the ACE-III cognitive scale. There were 35 (55.6%) participants in the non-cognitive impairment group, including 19 males and 16 females. The average age was 26.11 ± 8.105 years, and the average years of education was 12.20 ± 2.939 (Table 2). Thirty (47.6%) patients had abnormal gait with a st-TUG completion time ≥ 10 s. There were 32 (50.8%) cases of neurological type and 31 (49.2%) cases of hepatic type. The incidence of neurological symptoms in 32 WD patients with neurological disorders included dysarthria (18, 56.3%), dystonia (12, 37.5%), tremor (8, 25%), Parkinson’s-like (3, 9.4%) and chorea (3, 9.4%).

**Table 2:** Comparison of general data, scores of POMA, BBS, and bMRIsc-WD.

Group	*n*	Gender (male/female)	Age (years)	Years of education (years)	POMA	BBS	bMRIsc-WD
Cognitive impairment	28	10/18	29.89 ± 6.884	10.96 ± 2.380	25.39 ± 2.455	52.89 ± 2.378	3.93 ± 2.418
Non-cognitive impairment	35	19/16	26.11 ± 8.105	12.20 ± 2.939	27.17 ± 1.723	54.63 ± 1.716	1.66 ± 2.300
*t*		0.330	1.132	1.855	−3.374	−3.362	3.786
*p*		0.743	0.262	0.068	0.001	0.001	0.000

### Comparison of general data, POMA, BBS, and bMRIsc-WD

The POMA, BBS, and bMRIsc-WD scores of the cognitive impairment group were lower than those of the non-cognitive impairment group, and the difference was statistically significant (*p* < 0.05). This showed that the cognitive impairment group was worse than the non-cognitive impairment group in motor balance, gait stability, and brain lesion severity (Table 2). Comparison of total score, attention, memory, language fluency, language, and visual space in ACE-III between the cognitive impairment group and the non-cognitive impairment group of WD patients (Table 3).

**Table 3:** Comparison of each factor score of ACE-III.

Group	*n*	ACE-III score					
		Total score	Attention	Memory	Language fluency	Language understanding	Visual space
Cognitive impairment	28	78.21 ± 8.23	14.89 ± 2.21	18.88 ± 3.40	8.93 ± 2.05	21.96 ± 2.15	12.09 ± 2.12
Non-cognitive impairment	35	94.28 ± 3.52	18.14 ± 1.25	23.33 ± 1.51	13.54 ± 0.95	24.02 ± 1.37	16.18 ± 0.83
*t*		8.595	6.031	6.603	11.031	1.907	8.892
*p*		0.000	0.000	0.000	0.000	0.063	0.000

### Distribution of brain lesions on MRI

Brain MRI (bMRI) was abnormal in 39 (61.9%) patients showed brain MRI (bMRI) abnormalities from 63 WD patients. The lenticular nucleus (39/39, 100%) was the most commonly lesion site being involved, the frequency followed by brain atrophy (31/39, 79.5%), thalamus (20/39, 51.3%), caudate nucleus (17/39, 43.6%), brain stem (15/39, 38.5%). Signal abnormalities were also noted: corpus callosum (3/39, 7.7%), encephalomalacia (3/39, 7.7%) and cerebellum (2/39, 5.1%).

### Comparison of ACE-III, BBS, and POMA

Comparison of factor score of total score, language fluency, language understanding, visual space of ACE-III, BBS, and POMA, it was found that the group with bMRI abnormalities were less than the normal bMRI group in WD patients (*p* < 0.05) (Table 4).

**Table 4:** Comparison of ACE-III, BBS, and POMA between the normal group and the abnormal group on MRI.

Group	*n*	POMA	BBS	ACE-III score					
				Total score	Attention	Memory	Language fluency	Language understanding	Visual space
bMRI normal	24	27.42 ± 1.44	54.96 ± 1.57	91.75 ± 6.09	17.50 ± 0.93	23.79 ± 2.93	10.63 ± 2.12	24.25 ± 1.80	15.79 ± 0.42
bMRI abnormal	39	25.74 ± 2.43	53.18 ± 2.27	87.31 ± 5.92	17.38 ± 1.07	23.36 ± 2.37	9.36 ± 1.94	22.82 ± 2.30	14.51 ± 1.99
*t*		−3.434	−3.667	−2.842	−0.451	−0.611	−2.375	−2.745	−3.888
*p*		0.001	0.001	0.007	0.654	0.545	0.022	0.008	0.000

### Comparison of TUG gait performance parameters with different cognitive–motor dual tasks

Compared with the non-cognitive impairment group, the cognitive impairment group had a slower pace and shorter step size when completing the st-TUG, dt-TUG1, and dt-TUG2, and the differences were statistically significant (*p <* 0.05). At the completion of the dt-TUG2, there was no statistical difference in pace frequency between the two groups (*p* > 0.05). There was no difference in DTC values between cognitive impairment group and non-cognitive impairment group when performing dt-TUG number calculation and animal naming respectively (*p* > 0.05). However, regardless of cognitive impairment or not, the DTC2 values of number calculation tasks is higher than DTC1 of animal naming tasks in dt-TUG (*p* < 0.05). The one-way ANOVA showed that, compared with gait parameters under three different cognitive and motor dual-tasks, there were significant differences in pace speed and step size (*p <* 0.05). The cognitive impairment group had no significant difference in pace frequency (*p* > 0.05) (Table 5). This showed that different cognitive and motor dual-task participate in influencing the different gait performance parameters of WD patients.

**Table 5:** Comparison of performance parameters of different TUG tasks.

Gait parameters	TUG tasks	Cognitive impairment (*n* = 28)	Non-cognitive impairment (*n* = 35)	*t*	*p*
Pace speed	st-TUG	0.76 ± 0.043	0.89 ± 0.054	−10.245	0.000
	dt-TUG1	0.71 ± 0.026	0.84 ± 0.035	−16.487	0.000
	dt-TUG2	0.73 ± 0.031	0.86 ± 0.042	−13.620	0.000
	*F*	12.200	8.228		
	*p*	0.001	0.005		
Step size	st-TUG	0.50 ± 0.037	0.64 ± 0.051	−12.563	0.000
	dt-TUG1	0.45 ± 0.027	0.57 ± 0.038	−14.171	0.000
	dt-TUG2	0.46 ± 0.027	0.57 ± 0.040	−12.069	0.000
	*F*	17.190	44.900		
	*p*	0.000	0.000		
Pace frequency	st-TUG	92.67 ± 7.425	84.24 ± 8.636	4.092	0.000
	dt-TUG1	93.85 ± 6.143	87.84 ± 7.710	3.357	0.001
	dt-TUG2	95.16 ± 5.954	91.29 ± 9.131	1.936	0.058
	*F*	2.036	12.007		
	*p*	0.157	0.001		
DTC	DTC1	0.07 ± 0.041	0.06 ± 0.034	1.322	0.191
DTC2	0.06 ± 0.034	0.03 ± 0.020	1.114	0.270
	*t*	8.114	8.931		
	*p*	0.000	0.000		

### Correlation of gait parameters with cognitive domain factors

Pearman’s correlation coefficients was calculated to analyze the correlation between factor scores of ACE-III cognitive fields of WD patients and TUG gait parameters of different tasks. The results show that pace speed and step size are related to the total score, memory, language fluency, language understanding and visual space factor score of ACE-III (*p* < 0.05), but do not correlation with attention (*p* > 0.05). Pace frequency was correlated with memory and language comprehension factors (*p* < 0.05). There was no correlation between attention factor scores and TUG gait parameters of different cognitive and motor tasks (*p* > 0.05) (Table 6).

**Table 6:** The correlation analysis of gait parameters with cognitive domain factors (*r*).

Gait parameters	ACE-III score					
	Total score	Attention	Memory	Language fluency	Language understanding	Visual space
st-TUG pace speed	0.725**	0.224	0.526**	0.606**	0.475**	0.429**
st-TUG step size	0.719**	0.121	0.533**	0.558**	0.520**	0.422**
st-TUG pace frequency	−0.347	0.000	−0.259*	−0.236	−0.280*	−0.209
dt-TUG1 pace speed	0.786**	0.169	0.597**	0.588**	0.563**	0.491**
dt-TUG1 step size	0.747**	0.178	0.517**	0.582**	0.572**	0.426**
dt-TUG1 pace frequency	−0.317*	−0.107	−0.152	−0.276*	−0.290*	−0.138
dt-TUG2 pace speed	0.757**	0.176	0.560**	0.592**	0.536**	0.470**
dt-TUG2 step size	0.718**	0.234	0.486**	0.532**	0.522**	0.455**
dt-TUG2 pace frequency	−0.182	−0.155	−0.061	−0.106	−0.143	−0.138
DTC1	−0.060	0.143	−0.105	0.106	−0.138	−0.099
DTC2	0.005	0.213	−0.036	0.158	−0.132	−0.081

*p-value <0.05, **p-value <0.01.

### Correlation of cognitive domain factors and brain lesion areas

Multiple linear regression was used to analyze the correlations between the scores of cognitive factors in various lesional brain areas and severity. The scores of factors in various cognitive fields of the ACE-III were taken as dependent variables, and the brain areas involved in MRI lesions and the severity score of bMRIsc-WD were taken as independent variables. The results showed that brain atrophy was correlated with the total cognitive score, memory, and language comprehension factor scores of the ACE-III (*p* < 0.05), thalamic lesions were correlated with the total cognitive score of the ACE-III (*p* < 0.05), bMRIsc-WD severity score was correlated with language fluency and visuospatial factor scores (*p* < 0.05), and caudate nucleus and cerebellar lesions were correlated with visuospatial factor scores (*p* < 0.05) (Table 7).

**Table 7:** Regression analysis of ACE-III cognitive factors and related factors of lesional brain areas and bMRIsc-WD (stepwise).

ACE-III cognitive factors	Correlative factor	*B* value	Standard error	*t*	*p*
Total score	(constant)	93.010	1.001	92.939	0.000
	Brain atrophy	−6.103	1.343	−4.545	0.000
	Thalamus	−3.658	1.613	−2.267	0.027
Language fluency	(constant)	10.603	0.357	29.703	0.000
	bMRIsc-WD	−0.285	0.096	−2.965	0.004
Language understanding	(constant)	24.367	0.369	66.038	0.000
	Brain atrophy	−1.912	0.510	−3.751	0.000
Visual space	(constant)	15.772	0.256	61.689	0.000
	bMRIsc-WD	−0.203	0.075	−2.704	0.009
	Caudate nucleus	−2.422	0.858	−2.822	0.006
	Cerebellum	−1.802	0.779	−2.312	0.024

### The correlation of gait parameters with brain lesion areas

The gait performance parameters of the st-TUG, dt-TUG1, and dt-TUG2 were taken as dependent variables and different brain lesion areas were taken as independent variables to be included in the multiple linear regression analysis (stepwise method) (Table 8). The results showed that brain atrophy was related to the pace speed of the st-TUG and dt-TUG (*p* < 0.01), the lenticular nucleus was related to the step size of the st-TUG and dt-TUG (*p* < 0.01), and the pace frequency of the st-TUG, dt-TUG1, and dt-TUG2 were related to the thalamus, midbrain, and caudate nucleus, respectively (*p* < 0.01).

**Table 8:** Regression analysis of TUG performance parameters and brain lesion areas (stepwise).

Gait parameters	Dependent variable	Correlative factor	B value	Standard error	*Beta*	*t*	*p*
st-TUG	Pace speed	(constant)	0.863	0.013		65.005	0.000
		Brain atrophy	−0.058	0.019	−0.361	−3.024	0.004
	Step size	(constant)	0.612	0.014		44.129	0.000
		Lenticular nucleus	−0.071	0.019	−0.422	−3.636	0.001
	Pace frequency	(constant)	86.306	1.207		71.479	0.000
		Thalamus	8.131	2.658	0.365	3.059	0.003
dt-TUG1	Pace speed	(constant)	0.810	0.011		71.672	0.000
		Brain atrophy	−0.066	0.016	−0.457	−4.008	0.000
	Step size	(constant)	0.550	0.011		48.362	0.000
		Lenticular nucleus	−0.059	0.016	−0.426	−3.675	0.001
	Pace frequency	(constant)	88.662	1.229		72.154	0.000
		Midbrain	4.304	1.877	0.282	2.293	0.025
dt-TUG2	Pace speed	(constant)	0.834	0.012		69.902	0.000
		Brain atrophy	−0.063	0.017	−0.420	−3.616	0.001
	Step size	(constant)	0.548	0.011		51.390	0.000
		Lenticular nucleus	−0.050	0.015	−0.393	−3.338	0.001
	Pace frequency	(constant)	92.370	0.979		94.323	0.000
		Caudate nucleus	13.422	4.488	0.358	2.991	0.004

## Discussion

Previous studies have shown that the incidence of abnormal gait in WD patients ranges from 10% up to 75% (7, 20). This study showed that the incidence of gait abnormality and cognitive impairment in WD patients was 47.6 and 44.4%, respectively. Cognitive impairment in WD patients can be manifested in many cognitive fields of the ACE-III except language understanding. However, gait abnormality was related to many cognitive fields in the ACE-III except attention; cognitive influence on gait was mainly reflected in the change of pace speed and step size. This result is inconsistent with the study on the relationship between cognition and gait in patients with Parkinson’s disease that found that the pace and stride length of patients with cognitive impairment were linked to calculation, visual space, and attention (21), which may be related to the different pathogenesis of the two diseases. In patients with mild cognitive impairment, the time and spatial parameters of gait are decreased compared with the normal cognitive population, suggesting that stride length, swing speed, stride width, and pace speed can be used to determine cognitive impairment early (22). It can be seen that gait cannot be simply regarded as a simple action that includes a series of repetitive movements, but that this action also needs one or more cognitive processes, among which executive function is an important cognitive resource for normal movement, involving cognitive and behavioral components required for goal-oriented and successful actions, and is the basis for managing daily activities (23).

In WD, excess copper is also released into the circulation with resultant pathological accumulation in other tissues, particularly the brain, which can lead to neurological symptoms and psychiatric disturbances. Symptoms vary widely and present most commonly between ages 5 and 35 years (1). For a long time, the clinical manifestations of WD patients have been divided into two types: neurologic type was defined as WD in which neurological and/or psychiatric symptoms were initially present; and hepatic type was defined as presenting without neurological system involvement (2). Studies have confirmed that not only patients with neurological WD with neurological symptoms but also hepatic patients with liver cirrhosis may also have cognitive impairment, including executive function and cognitive flexibility (24–26). The results of this study showed that there were 32 (50.8%) patients with the neurologic type, 28 (44.4%) patients with cognitive impairment, and 39 (61.9%) patients with abnormal brain MRI, which reflected the incomplete matching of cognitive impairment, neurologic type, and abnormal brain MRI. Therefore, it has been suggested that the current binary clinical phenotype classification method of WD may no longer be suitable for cognitive impairment (27). In addition, this research showed that WD patients with cognitive impairment were worse than those with non-cognitive impairment in motor balance and gait stability (*p* < 0.05). Thus, cognitive impairment in WD patients affected gait parameters, especially gait speed and stride length, which were mainly affected by many cognitive domains other than attention, and not just memory, language fluency, language understanding, visual space and other cognitive areas. Under different cognitive motor tasks, WD patients with cognitive impairment showed pace speed and step size <javascript:%20void(0)> (*p* < 0.05), but not pace frequency (*p* > 0.05). It can be seen that gait is also an outward manifestation of the overall cognitive impairment of WD patients, and the cognitive impairment of WD patients can be predicted by the gait performance parameters of pace speed and step size in the clinic.

The 3-meter TUG test is a standard examination test used in clinical practice to detect gait and ambulatory function; adding a cognitive dual tasking component to the TUG test has been shown to further improve the detection of fall risk in this population, as distinct cognitive correlates relate to different types of dual tasking costs seen on the TUG (28). The cognitive contribution to gait control is supported by experimental evidence offered by the dual task paradigm, including both the “absolute” performance during the dual task and the interference that expresses the change on walking between dual task and single task performance (29). In this study, our results demonstrated that different cognitive motor dual tasks participated in influencing the different gait performance parameters of WD patients. Under different cognitive motor tasks, WD patients with cognitive impairment showed gait speed and stride length (*p* < 0.05), but not gait frequency (*p* > 0.05). This shows that different cognitive-motor dual tasks participate in influencing different gait performance parameters of WD patients. In addition, it was found that the factor scores of each cognitive domain of the ACE-III score were not correlated with DTC1 and DTC2 (*p* > 0.05). This shows that the overall DTC of the complete TUG is related to the overall cognitive impairment, but not to any discrete cognitive subdomain test. Cognitive motor interference theory holds that walking while performing dual tasks requires high-level cognitive control in the execution process and attention ability, and its damage may lead to the degradation of motor and cognitive execution. Dual tasks in walking are very common in everyday life and affect gait and cognitive performance, which depends on age, attention priority, task complexity, and medical conditions (30).

DTC is utilized to study the interaction between exercise and cognition in the exercise–cognitive dual task paradigm, as a measure of the effect of dual tasks under physiological and pathological conditions (18). This study showed that there was no difference in the DTC value of dual task consumption between WD patients with cognitive impairment and patients with non-cognitive impairment when performing dt-TUG number calculation and animal naming (*p* > 0.05), but both groups had higher DTC values of dual-task consumption when performing dt-TUG number calculation task compared with the animal-naming task (*p* < 0.05). It has been shown that the cognitive task of digital computing is more sensitive than animal naming in evaluating the cognitive impairment of WD patients, which further proves that DTC depends on the complexity of the cognitive task, not on age and disease (31). Based on this study, it is found that cognitive impairment of WD patients affects gait, especially when performing cognitive motor dual tasks. It can be seen that dual task training can improve walking ability and balance ability, which is more effective than sequential training in patients with different brain injuries, because we will perform static or dynamic tasks in daily life (32, 6). Therefore, the design of different cognitive and motor dual-task training for WD patients may help to further promote the rehabilitation of patients with abnormal gait, which is worth further investigation.

WD may present as liver or neuropsychiatric symptoms, cognitive-related neuroimaging studies have shown that a neural phenotype characterized by subtle cognitive impairment in WD patients is caused by diffuse changes in gray matter and white matter of cortex, while a more obvious neural phenotype characterized by dyskinesia and executive dysfunction appears with the deterioration of basal ganglia lesions (26). In a cross-sectional study, even in patients with WD with an asymptomatic nervous system, there are changes in brain structure and function (33), brain MRI changes may be present even in patients without neurological symptoms, brain atrophy is also evident in hepatic type WD without neurological symptoms (34, 35). Therefore, it is difficult to define whether cognitive impairment exists through clinical classification, in order to evaluate cognitive impairment of WD patients more comprehensively and objectively, our study did not use the commonly binary clinical phenotype classification with the hepatic and neurologic type.

Typical imaging manifestations on MRI in WD patients are cerebral atrophy, cerebellum, white matter of the cerebral hemispheres and symmetrical hyperintensity or mixed intensity on T2-weighted images changes in basal ganglia, thalamus, and brainstem. A series of extrapyramidal symptoms, such as Parkinson’s-like symptoms, tremors, and dystonia, may affect gait (36). The results of this study show that brain atrophy, the thalamus, caudate nucleus, and cerebellum were correlated with cognitive impairment (*p* < 0.05). The lenticular nucleus was related to step size, brain atrophy was related to pace speed, and the thalamus, caudate nucleus, and midbrain were involved in the step frequency in WD patients (*p* < 0.05). Some studies believe that the quantitative measurement of gait is helpful to confirm that patients with WD have long-term persistent gait abnormalities and the risk of frequent falls, especially those with the neurologic type, and it is more obvious with the extension of the course of the disease, the study emphasizes the positive influence of early diagnosis and prompt treatment on prognosis, especially on the persistence of abnormal gait in WD patients (3, 36). It can be seen that cognitive impairment can affect gait, and the associated participation of cognitive impairment and pathological brain regions may be the principal neural mechanism of gait abnormality in WD patients. Therefore, for the clinical intervention of gait disorder, in addition to copper chelation therapy to promote the reversible recovery of pathological brain atrophy, the lenticular nucleus, and other brain regions, cognitive and motor dual-task training may need to be strengthened.

At present, the pathomechanism of the influence of cognitive impairment on gait abnormality is not completely clear, which needs to be confirmed by larger prospective studies. Because the clinical presentation of WD includes hepatic and neuropsychiatric manifestations, the factors affecting cognitive impairment are complex and diverse. In this cognitive and motor dual-task study, we initially discussed the effect of cognitive impairment on gait abnormalities in WD patients, and different cognitive and motor dual-task were involved in affecting gait parameters. However, this study also has some limitations. In the pathological mechanism, the exact lesion brain regions involved in cognitive impairment and the possible effects of cirrhosis and mild hepatic encephalopathy on cognition and gait deserve further exploration and study.

## Conclusion

WD patients had a high incidence of cognitive impairment and gait disorder, the pace speed and step size can reflect the cognitive impairment of WD patients, cognitive impairment affects the gait disorder of WD patients, and the different cognitive and motor dual-tasks were involved in affecting gait parameters. The joint participation of cognitive impairment and lesion brain area may be the principal neural mechanism of gait abnormality in WD patients.

## Data availability statement

The original contributions presented in the study are included in the article material, further inquiries can be directed to the corresponding author.

## Ethics statement

The studies involving humans were approved by the Affiliated Hospital of Institute of Neurology, Anhui University of Traditional Chinese Medicine. The studies were conducted in accordance with the local legislation and institutional requirements. The participants provided their written informed consent to participate in this study. Written informed consent was obtained from the individual(s) for the publication of any potentially identifiable images or data included in this article.

## Author contributions

GW and YH contributed to the formulation or evolution of overarching research goals and aims. PJ, XM, KL, and LZ played a role in the plan implementation, wrote, and edited the manuscript. XH, XW, and XB contributed to the investigation process, specifically handling the experiments, and data collection. All authors contributed to the article and approved the submitted version.

## Funding

This study was supported by the Major Project of Clinical Research Fund of Anhui University of Traditional Chinese Medicine (2021sfylc04) and the 2023 Scientific Research Fund Project of Colleges and Universities in Anhui Province. The funders had no role in study design, data collection, and analysis, decision to publish, or preparation of the manuscript.

## Conflict of interest

The authors declare that the research was conducted in the absence of any commercial or financial relationships that could be construed as a potential conflict of interest.

## Publisher’s note

All claims expressed in this article are solely those of the authors and do not necessarily represent those of their affiliated organizations, or those of the publisher, the editors and the reviewers. Any product that may be evaluated in this article, or claim that may be made by its manufacturer, is not guaranteed or endorsed by the publisher.
